# ALDH1 Cancer Stem Cell Marker as a Prognostic Factor in Triple-Negative Breast Cancer

**DOI:** 10.1155/2020/7863243

**Published:** 2020-07-03

**Authors:** Sonar Soni Panigoro, Dian Kurnia, Ahmad Kurnia, Samuel Johny Haryono, Zafiral Azdi Albar

**Affiliations:** ^1^Oncology Division Department of Surgery Cipto Mangunkusumo National Hospital, Faculty of Medicine Universitas Indonesia, Jakarta 10430, Indonesia; ^2^Department of Surgical Oncology, Dharmais Hospital, National Cancer Hospital, Jakarta 11420, Indonesia

## Abstract

Breast cancer is the most common cancer with an increasing incidence in Asia. About 20% of all breast cancers are triple-negative breast cancers (TNBCs). BCSC is a subset of tumor cells that has stem cell-like characteristics, such as a high capacity for self-renewal and tumor initiation, which implies that BCSC may cause aggressiveness of TNBC. ALDH1 has a role in early stem cell differentiation through its function in the oxidation of retinol to retinoic acid, proposed to be a strong candidate for breast cancer stem cells. Various studies have shown that ALDH1 is one of the markers of CSC that can be used as a prognosis indicator because it can be a biological marker for poor prognostic factors in TNBC. This study assessed the prognostic survival rate with a retrospective cohort method in TNBC patients. A total of 54 of 55 patients treated at RSCM were tested for the expression of ALDH1 through an immunohistochemistry assay of breast cancer tissue using ALDH1 staining. Survival analysis was done to obtain the prognostic data of ALDH1. Positive ALDH1 expression was obtained at 38.89% in TNBC patients. One-year survival and three years of survival in TNBC patients with positive ALDH1 expression were 42.9% and 33.3%, respectively. In this study, ALDH1 can be used as a poor survival prognostic factor with HR 2.636 and *p* value 0.013. The conclusion of this study is that ALDH1 can be used as a poor prognostic factor in TNBC patients although it cannot be an independent prognostic factor.

## 1. Background

Breast cancer is the most common cancer with an increasing incidence in Asia. According to GLOBOCAN 2012, the incidence of breast cancer in Indonesia was 40.31 per 100,000 women, with a death rate of 16.58 per 100,000 women. The five-year survival rate of patients with a diagnosis of localized, regionally metastasized, and remotely metastasized breast cancers were 98.5%, 84.6%, and 25%, respectively [1].

There are various types of tumors in breast cancers, which also possess different characteristics, clinical outcomes, and therapeutic responses. About 20% of all breast cancers are triple-negative breast cancers (TNBCs) because they contain little estrogen and progesterone receptors and lack the HER2 gene. TNBC also has an aggressive clinical course including high metastasis to the visceral organs and central nervous system [2]. TNBC has a higher prevalence in African Americans, more frequently affects younger patients (average age <50 years), and is associated with a greater risk of mortality [3].

A study in Brazil shows that the overall survival was 62% for TNBC and 81% for non-TNBC and disease-free survival was 57% for TNBC and 75% for non-TNBC. This tumor subtype tended to show a worse clinical course, with earlier and more frequent recurrence and worse 5-year survival, compared with non-TNBC [4]. Aside from having a poor prognosis, triple-negative breast cancers also pose a challenge in terms of therapy. Therefore, new biologic markers for prognosis, including prediction of therapeutic resistance, are needed. Among others are the breast cancer stem cells (BCSCs), which are believed to have a contribution to tumorigenesis, metastasis, and resistance against chemotherapy. Breast cancer stem cell is a subset of tumor cells that has a characteristic of stem cells, having a high capacity of self-renewal and tumor initiations, which imply that BCSC may cause aggressiveness of TNBC [5].

The majority of the biologic markers that are proven to have an association with TNBC resistance against therapy are found in the cytoplasm of TNBC cells. Identification of cytoplasmic biologic markers in breast cancers, which are proteins that run in the PUKCA/AKT/mTOR pathways, such as PIK3CA, PTEN, pAKT/pS6, metabolites, and aldehyde dehydrogenase 1 (ALDH1), is being widely developed [3].

ALDH1, which has a role in the early differentiation of stem cells through its function in the oxidation of retinol to retinoic acid, is suggested to be a strong candidate for cancer stem cells in the breast. Various studies showed that ALDH1 is one of the CSC markers that can be used as an independent prognostic indicator in node-positive breast cancer [6]. There has been no study of ALDH1 as a prognostic indicator for TNBC in Indonesia; therefore, this research is essential to be made. From this study, it is hoped that we can determine whether ALDH1 cancer stem cell markers are a poor prognostic factor in triple-negative breast cancer patients. Therefore, we can determine survival rates for triple-negative breast cancer patients with ALDH1.

## 2. Methods

### 2.1. Study Design

This study was a retrospective cohort with a unique design of survival analysis. Data were obtained retrospectively from the medical records of the Oncology Surgery and Anatomical Pathology departments at Cipto Mangunkusumo National General Hospital (RSCM), and then the history of recurrence and mortality were recorded.

### 2.2. Sample

The accessible population was patients who were firstly diagnosed with triple-negative breast cancers from January 2010 to December 2016 who came to RSCM, with an observation period until December 2017. The sampling method is done by performing consecutive sampling, using the sample size formula, and the sample size (*n* = 55) was obtained. The sample that is used is all patients who came to the oncology surgical clinic, who met the inclusion criteria. The sampling method was carried out by obtaining the data of paraffin blocks from the anatomical pathology laboratory. All subjects who met the inclusion criteria were included in the study until the sample size was fulfilled.

Inclusion criteria were specimens of triple-negative breast cancer paraffin blocks after immunochemistry examination was made, the specimens able to be adequately stained with ALDH1 immunochemistry staining, well-recorded medical records, and patients with triple-negative breast cancers who already had therapy, whereas exclusion criteria included paraffin blocks that were considered broken or missing, broken specimens upon staining, and incomplete medical records.

### 2.3. Variable and Data Measurements

Data from medical records of patients with triple-negative breast cancers from January 2010 to December 2016 were determined at a follow-up of three years since the diagnosis and were treated until the completion of the study. From the medical records, data regarding identities, epidemiological data, clinical data and anamnesis, physical examinations, tumor size, clinical staging, and supporting examinations on the patients' first visit were obtained. The anatomical pathology registration number was recorded. From the registration number, data about histopathological diagnosis, grading, and Ki67 were obtained. Thereafter, immunohistochemistry staining for ALDH1 was done on paraffin blocks from the specimens of tumor tissue biopsy.

From the results of the staining specimens and ALDH1 levels, which are independent variables, a sample group was divided into positive ALDH1 groups who had died, positive ALDH1 who had survived, negative ADLH1 who had died, and negative ADLH1 who had survived. After group division, survival analysis is carried out to determine the survival rate, as a dependent variable, with the hope of determining the survival rate for one year and three years.

In this study, sampling was not determined by age limits or specific criteria. Instead, we determined several variables that could influence the determination of prognostic factors in triple-negative patients, such as age (>50 years or <50 years), tumor size (T1, T2, T3, or T4), lymph node metastases (N0, N1, N2, or N3), distant metastases (present or not), histological grading (based on the Bloom and Richardson criteria), Ki67 (<20% or > 20%), local recurrence (whether or not there is), and chemotherapy therapy that has been given until before the study is conducted (NO and CEF/CAF) for analysis between variables with survival rates.

### 2.4. Statistical Methods

After data were collected, we conducted a descriptive analysis to determine the characteristics of research subjects. Then, survival analysis using the Kaplan–Meier method is performed to show the overall survival rate. Bivariate analysis was also performed between ALDH1 expression and survival rate (*p* < 0.05), as well as analyzing the survival of the negative ALDH1 and positive ALDH1 groups using the Kaplan–Meier method. In the presence of confounding variables, an analysis of the relationship between these variables and survival rate was performed. From these results (*p* < 0.05), a multivariate analysis was performed.

### 2.5. Ethics Study

This study did not involve patients directly, and the sample that we used was a paraffin block. This study was approved by the Faculty of Medicine, Universitas Indonesia Ethics Committee, with number 1072/UN2.F1/ETIK/2017.

## 3. Results

There were 367 patients with TNBC who were admitted to the oncology surgery department at RSCM from 2010 to 2016. Based on the inclusion and exclusion criteria, fifty five samples were obtained. One sample was excluded due to damage to the paraffin block. Therefore, a total of 54 samples participated in this study. Twenty four subjects (44.44%) experienced an event of interest, which was death due to any reasons, and thirty subjects (55.56%) were alive until the end of the observation period. The observation period had a median of 21 months with an interval of 1 to 79 months.

Most of the subjects in this study were under the categories of younger than 50 years (53.70%), T4 tumor size (44.44%), involvement of the N1 lymph node (57.41%), no metastasis (72.22%), grade 2 histological grading (51.84%), Ki67 ≥ 20% (83.33%), stages 2 and 3 (35.19%), chemotherapy TC (48.15%), no recurrence (77.78%), radiotherapy (57.41%), and negatively expressed ALDH1 (61.11%) (Table 1).

**Figure 1:**
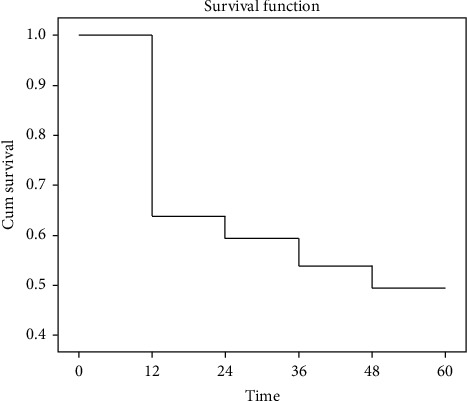
Kaplan–Meier curve for overall survival.

The survival analysis with the Kaplan–Meier method showed an overall survival rate in 12, 36, and 60 months as in Figure 1. From the bivariate analysis, there was an association between the expression of ALDH1 with the survival rate, with HR 2.636 (95% CI 1.168–5.950), *p*=0.013 (Table 2). Besides, there was also a significantly different survival rate between negatively and positively expressed ALDH1 against the survival rate of TNBC (log-rank *p*=0.013), where the negative ALDH1 group had a longer survival rate compared to those with positive ALDH1. Negative ALDH1 had a lifespan of 43 months, where positive ALDH1 had a lifespan of 24 months (Table 2).

**Figure 2:**
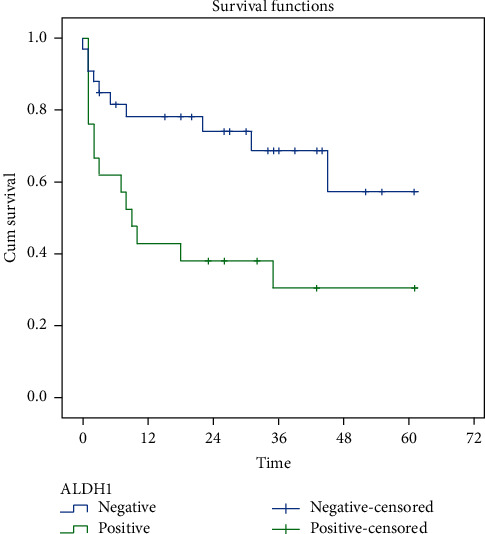
Kaplan–Meier curve which shows the survival rate of ALDH1.

From the Kaplan–Meier survival analysis, it can be seen that at the beginning of the follow-up, the proportion between positive ALDH1 expressions occurred significantly in the first year, whereas there were 12 deaths in the first year on positive ALDH (Figure 2).

The observation on the relationship between variables with the increase in the hazard ratio showed that the factors which had a significant proportion of the hazard ratio were T4 tumor size (HR 6.94 [95% CI 2.02–6.86] with *p*=0.002), remote metastasis (HR 14.89 [95% CI 5.91–37.5] with *p* < 0.001), advanced stage (HR 38.31 [95% CI 8.25–177.95] with *p* < 0.001), and other types of chemotherapy (HR 3.42 [95% CI 1.190–10.61] with *p*=0.033). The Ki67 ≥ 20% group had HR 1.24 (95% CI 0.422–3.630); however, it was not statistically significant (*p*=0.697) (Table 3).

Variables that have *p* value <0.05 in the bivariate analysis are included in multivariate analysis. Variables included in multivariate analysis were tumor size, distant metastases, staging, and chemotherapy. Nevertheless, based on the concept of collinearity, where tumor size and metastasis are part of the stadium, only stadium and chemotherapy are included in multivariate analysis. From the multivariate results, it was found that HR reduced from ALDH 1 to 0.982. Changes in the adjusted hazard ratio for positive ALDH1 expression at each addition of confounding variables can be seen in Table 4.

## 4. Discussion

The age range of patients in this study was 25 to 75 years, with an average age of 50.9 years. According to the patient characteristic data, twenty nine patients (53.70%) aged <50 years and 25 patients (46.30%) aged ≥50 years. The highest number of mortality was found in the age group of <50 years, with a percentage of 25.9%. The risk of patients aged ≥50 years was found to be 0.7 times less than those aged <50 years. However, the result was not considered to be significant since the *p* value was 0.535.

Regarding tumor size, a significant result was found in T4 tumor size compared to T1-T2 tumor size (*p*=0.002). The group with T4 size of the tumor appeared to have 6.94 times greater risk than the group with T1-T2 size of the tumor. However, the group with T3 size of tumor possessed 0.859 hazard ratio. However, the result was not significant (*p*=0.868). It was consistent with the study by Ma et al., which showed that the result of overall survival was 1.253 times higher risk in tumor >2 cm than tumor <2 cm; however, it was not significant (*p*=0.647). The difference in this study was that the grouping of tumor size was based on the TNM criteria [7]. A significant result was also found in the group with remote metastases than those without metastases, with a risk of 14.98 times (*p* < 0.001).

A significant result was found in terms of staging, which showed that the advanced stage group possessed 38.309 times higher risk compared to the early-stage group (*p* < 0.001). The result of a locally advanced stage group revealed to have 4.544 times greater risk compared to the early-stage group; however, it was not statistically significant (*p*=0.059). This finding was consistent with the study by Ma et al., which showed 5.511 times greater risk at stage III compared to stage I-II with a *p* value of 0.033 [7].

In terms of chemotherapy, the group that received other types of chemotherapy had 3.422 times higher risk than those with TC chemotherapy. This result was statistically significant (*p*=0.033). Besides, the group which acquired CAF/CEF chemotherapy was found to have 1.741 times greater risk compared to the group with TC chemotherapy. However, this result was not statistically significant, with the *p* value of 0.235.

The expression of positive ALDH1 was 38.89% from a total patient with TNBC in this study. This finding did not differ much from the study by Perou et al. (31.6%), Yoshioka et al. (26%), and Zhou et al. (35%) [6, 8–10].

The expression of ALDH1 was found to be statistically significant concerning survival rate and HR with a result of 2.636 (*p*=0.013). This result showed that TNBC samples with positive ALDH1 possessed 2.636 times higher risk compared to those with negative ALDH1. The survival time of patients with positive ALDH1 and negative ALDH1 was 24 months and 43 months, respectively. As described in the Kaplan–Meier graph (Figure 2), the significant number of mortality happened in the first year of those with positive ALDH1 expression. It was consistent with the study by Ma et al. who found that TNBC with positive ALDH had a 2.368 times risk compared to negative ALDH [7]. The result was statistically significant with a *p* value of 0.039. Similar results were also suggested by Zhou et al. (HR 19.186), Yoshioka et al. (HR 1.1930), Zhong et al. (HR 11.932), and Dewi, who stated that positive ALDH1 expression suggested poor prognosis in patients with TNBC [6, 9–12].

Ohi et al. stated that several studies had reported identifying aldehyde dehydrogenase 1 (ALDH1) as the marker of the cancer stem cell, which was clinically significant in indicating the prognosis of patients with breast cancer. They also found the prognosis of TNBC with positive ALDH1 to be reduced. Besides, patients who experienced nodal metastases with positive ALDH1 expression were also suggested to have poor prognosis. They also found that TNBC expresses ALDH1 more often than the other types of breast cancer [13].

Research by Zhou et al. which studied the level of ALDH1 and other factors measured with immunohistochemistry staining, also supported those results. Zhou et al. revealed that ALDH1 was higher in cases with TNBC compared to non-TNBC cases (*p*=0.015). CC3 staining and positive ALDH1 were significantly correlated with poor prognosis of breast cancer with the TNBC subtype. Besides, positive ALDH1 was also related to reduced overall survival rate (RR = 2.83; 95% CI 2.16–3.67; *p*=0.001). Stem cell marker was a prognostic factor in breast cancer [9].

Poor prognosis influenced by ALDH1 was described in the metabolic pathway of PIK3CA/AKT/mTOR of breast cancer concerning grading and metastases. ALDH1 might be suggested as the specific biomarker for TNBC in the future direction. Studies also identified a lower survival rate with positive ALDH1 expression. ALDH1 catalyzes endogenous and exogenous aldehyde oxidation into an inactive carboxylic acid. ALDH1 is known to be a cytoplasmic stem cell-related marker that was found in breast cancer, and it was associated with tumor initiating cells. ALDH1 expression is significantly correlated with tumor grade metastasis, and it may be associated with the enhancement of taxane- and epirubicin-based chemotherapy resistance [3].

According to the other study by Zhong, positive ALDH1A1 cells were detected in 93 out of a total of 147 tumors (63.3%). Besides, eighty percent (32 of 40) tumors with strong ALDH1A1 staining showed early recurrence compared to 20.0% (8 of 40) tumors with negative ALDH1A1 expression (*p*=0.027). The ALDH1A1 correlated significantly with malignant proliferations based on Ki67 staining (*p*=0.001), indicating an association of the ALDH1A1 phenotype with malignant proliferation in invasive ductal carcinoma [11].

Moreover, Yoshioka et al. mentioned ALDH1 as the detoxification agent, which oxidized intracellular aldehyde and caused the resistance of the alkylating agent. The fact that ALDH1 possessed the ability to carry out detoxification by protecting the stem cell from the influence of oxidation explained the low response of therapy. Besides, ALDH1 also played a role in the conversion of retinol to retinoic acid, which acted as the stimulus of stem cell proliferation. Thus, it resulted in the low survival rate of TNBC with positive ALDH1 expression [6]. Zhou et al. supported those findings by stating that positive ALDH1 expression was correlated with the resistance of chemotherapy and poor prognosis [9].

Through the *chi-square* test and multivariate analysis, this study found that the clinicopathological factors, which influenced the statistical analysis of ALDH1 and survival rate, were the size of the tumor, advanced metastasis, advanced stage, and chemotherapy. Yoshioka et al. found that large size tumors and high grades were associated with ALDH1 expression [6]. Also, Ginister et al. mentioned the relationship of ALDH1 expression with high grade and poor OS, and Ohi et al. stated the association of high grade with ALDH1 expression [13]. Overall, almost all references in this study proposed that ALDH1 expression was correlated with high grade.

ALDH1 is one of the ethanol metabolism principal enzymes which equip the human breast epithelium, so a role comparison review with the other enzymes is needed. ADH, ALDH, and ADH isoenzyme expressions are lower in tumor cells than in the normal cells [14]. Thus, these enzymes are considered have important roles in carcinogenesis process. However, based on a study by Jelski W et al. on general breast cancer patients, the activity of ADH1 showed significantly highest difference in the serum of stage IV breast cancer patients [15]. Further research on ethanol metabolism enzymes is recommended to compare its activities in TNBC patients.

In this study, reduction in HR was obtained from multivariate analysis, and it can be concluded that ALDH1 cannot be an independent prognostic factor because it still depends on other variables. However, Zhong et al. suggested the ALDH1A1 phenotype to be an independent predictor of tumor recurrence in the early phase, especially in the event of early localized recurrence and advanced metastases of invasive ductal carcinoma [11]. However, another study that was held in Africa with patients from Ghana found an association between the prevalence of ALDH1 expression in TNBC and androgen receptor (AR) expression. It was found that AR-expressing cells in TNBC are more sensitive than AR antagonist cells. From these findings, it is known that ALDH1 can be a potential predictive biomarker for AR targeted therapy as well as a prognostic marker in TNBC [3].

Although there are significant results of this study, we suggest further research is needed, if necessary, multicenter research, to increase the number of samples and reduce confusion caused by certain variables.

## 5. Conclusion

In this study, we can conclude that the one-year survival rate for triple-negative breast cancer patients with positive ALDH1 expression was 42.9% and the one-year survival rate for triple-negative breast cancer patients with negative ALDH1 expression was 78.8%, also the three-year survival rate for triple-negative breast cancer patients with positive ALDH1 expression was 33.3%, and the three-year survival rate for triple-negative breast cancer patients with negative ALDH1 expression was 72.7%. From the statistical analysis of the data, ALDH1 can be used as a prognostic factor of poor survival in triple-negative breast cancer patients with a hazard ratio of 2.636 with a *p* value of 0.013 although it cannot be an independent prognostic factor.

## Figures and Tables

**Table 1:** Subject characteristics.

Variables	Total	Percentage
*Age*		
≥50 years	25	46.30
<50 years	29	53.70

*Tumor size (T)*		
1	2	3.70
2	14	25.93
3	14	25.93
4	24	44.44

*Involvement of lymph nodes (N)*		
0	15	27.78
1	31	57.41
2	4	7.41
3	4	7.41

*Remote metastasis (M)*		
Negative	39	72.22
Positive	15	27.78

*Histological grading*		
1	2	3.70
2	28	51.85
3	24	44.44

*Ki67*		
<20%	9	16.67
≥20%	45	83.33

*Staging*		
1	1	1.85
2	19	35.19
3	19	35.19
4	15	27.78

*Chemotherapy*		
TC	26	48.15
CAF/CEF	25	46.30
Others	3	5.56

*Recurrence*		
Negative	46	85.19
Positive	8	14.81

*Radiotherapy*		
Negative	23	42.59
Positive	31	57.41

*ALDH1*		
Positive	21	38.89
Negative	33	61.11

**Table 2:** Relationship between ALDH1 and survival rate.

Variables		Status				Mean survival rate (months)	HR (95% CI)	*p* value^*∗*^
		Alive		Dead				
		*n*	%	*n*	%			
ALDH1	Negative	14	66.67	7	33.33	43	2.636	**0.013**
	Positive	10	30.60	23	69.70	24	(1.168–5.950)	
Total		24	44.44	30	55.56			

**Table 3:** Relationship between all variables and survival rate.

Characteristics	Status				*HR* (IK 95%)	*p*
	Died		Live			
	*n*	%	*n*	%		
*Ages*						
≥50 years	10	40.00	15	60.00	Reff	
<50 years	14	48.28	15	51.72	1.294 (0.574–2.916)	0.535

*Tumor size*						
T1-T2	3	20.00	12	80.00	Reff	
T3	2	14.29	12	85.71	0.859 (0.142–5.143)	0.868
T4	19	76.00	6	24.00	6.935 (2.019–23.819)	**0.002**

*Lymph node metastasis*						
N0-N1	19	42.22	26	57.78	Reff	
N2	2	50.00	2	50.00	0.966 (0.223–4.175)	0.963
N3	3	60.00	2	40.00	2.020 (0.595–6.858)	0.260

*Distant metastasis*						
Negative	9	23.08	30	76.92	Reff	
Positive	15	100.00	0	0.00	14.894 (5.909–37.54)	**<0.001**

*Histological grading*						
1	1	50.00	1	50.00	Reff	
2	12	42.86	16	57.14	0.603 (0.078–4.669)	0,628
3	11	45.83	13	54.17	0.631 (0,081–4,936)	0.661

*Ki67*						
<20%	4	40.00	6	60.00	Reff	
≥20%	20	46.51	23	53.49	1.238 (0.422–3.630)	0.697

*Stadium*						
Early	2	10.00	18	90.00	Reff	
Advance local	7	36.84	12	63.16	4.544 (0.942–21.921)	0.059
Advance	15	100.00	0	0.00	38.309 (8.247–177.950)	**<0.001**

*Chemotherapy*						
TC	12	44.44	15	55.56	Reff	
CAF/CEF	8	38.10	13	61.90	1.741 (0.698–4.342)	0.235
Others	4	80.00	1	20.00	3.422 (1.103–10.610)	**0.033**

*Recurrence*						
Negative	21	45.65	25	54.35	Reff	
Positive	3	37.50	5	62.50	1.493(0.444–5.024)	0.517

*Radiotherapy*						
Negative	9	40.91	13	59.09	Reff	
Positive	15	48.39	16	51.61	0.811 (0.354–1.856)	0.620

**Table 4:** Crude HR and adjusted HR with 95% IK for ALDH1 expression for mortality in the gradual addition of confounding variables.

Variable expression ALDH	HR (IK 95%)	HR change (%)
*Crude HR*	2.636 (1.168–5.950)	

*Adjusted HR*		
+ Stadium	0.969 (0.373–2.157)	63.24
+ Chemotherapy	0.982 (0.373–2.157)	1.3

## Data Availability

The data used and analyzed during the study are available from the corresponding author upon request.
